# ToF-SIMS 3D Analysis of Thin Films Deposited in High Aspect Ratio Structures via Atomic Layer Deposition and Chemical Vapor Deposition

**DOI:** 10.3390/nano9071035

**Published:** 2019-07-19

**Authors:** Alireza M. Kia, Nora Haufe, Sajjad Esmaeili, Clemens Mart, Mikko Utriainen, Riikka L. Puurunen, Wenke Weinreich

**Affiliations:** 1 Fraunhofer Institute for Photonic Microsystems, 01099 Dresden, Germany; 2 VTT Technical Research Centre of Finland Ltd., 02044 Espoo, Finland; 3 School of Chemical Engineering, Aalto University, 02150 Espoo, Finland

**Keywords:** ToF-SIMS 3D imaging, compositional depth profiling, high aspect ratio (HAR) structures, silicon doped hafnium oxide (HSO) ALD deposition, lateral high aspect ratio (LHAR), ToF-SIMS analysis

## Abstract

For the analysis of thin films, with high aspect ratio (HAR) structures, time-of-flight secondary ion mass spectrometry (ToF-SIMS) overcomes several challenges in comparison to other frequently used techniques such as electron microscopy. The research presented herein focuses on two different kinds of HAR structures that represent different semiconductor technologies. In the first study, ToF-SIMS is used to illustrate cobalt seed layer corrosion by the copper electrolyte within the large through-silicon-vias (TSVs) before and after copper electroplating. However, due to the sample’s surface topography, ToF-SIMS analysis proved to be difficult due to the geometrical shadowing effects. Henceforth, in the second study, we introduce a new test platform to eliminate the difficulties with the HAR structures, and again, use ToF-SIMS for elemental analysis. We use data image slicing of 3D ToF-SIMS analysis combined with lateral HAR test chips (PillarHall™) to study the uniformity of silicon dopant concentration in atomic layer deposited (ALD) HfO_2_ thin films.

## 1. Introduction

Thin film deposition techniques such as atomic layer deposition (ALD), for three-dimensional microscopic structures and the development of large-scale integrated structures like trenches and cavities that are of importance, especially in the semiconductor industry, introduce a true challenge for thin film characterization. These 3D-micro-structured substrates have typically vertical high aspect ratio (VHAR) structures. The study and characterization of specific regions of interest like local defects, doping concentration, conformality, and interfaces, rely predominantly on cross-sectional sample preparation and characterization by electron microscopy or X-ray techniques. This approach faces the specific difficulties of single lamella preparation of each trench, low spatial resolution to characterize interfacial diffusion, or lack of detection of light elements in transmission electron microscopy (TEM) and cleavage plane inaccuracy for scanning electron microscopy (SEM).

To overcome the difficulties of the abovementioned characterization techniques, we used time-of-flight secondary ion mass spectrometry (ToF-SIMS) on HAR structure analysis. The SIMS technique is a powerful tool which provides information about a given material from both its surface, with an overall sensitivity of parts per million (ppm), and bulk, with a sensitivity of part per billion (ppb) [1,2]. The in-depth analysis of a thin film’s chemical composition, as well as interfacial characterization, yields information about the elemental distribution in the range of a few monolayers (1–3 monolayers). Furthermore, by using advanced liquid metal ion guns (LMIG), one can reach a lateral resolution of about 100 nm for the elemental detection (ToF-SIMS imaging) [3,4,5]. In addition to both remarkable in depth and lateral resolution, numerous parameters can affect the ToF-SIMS measurement technique that make data acquisition and interpretation challenging. For instance, the achievable spatial resolution in ToF-SIMS imaging is a function of the sample matrix, material concentrations, surface geometry, primary ion intensity, instrument transmission, and spot size of the primary ion beam [3,6,7].

In this paper, the first part of the study focuses on a non-copper-plated area at the bottom of large through-silicon-vias (TSVs). The TSV interconnects provide the shortest electrical pathway, lower power consumption, lower noise, smaller form factor, and yield better performance and more functionality in comparison with the conventional chip multi-layers stacking (CMLS) [8,9]. The most common TSV metallization stack is composed of a copper (Cu) diffusion barrier and a seed layer followed by the bottom-up being electroplated [10]. One of the main difficulties in the characterization of TSV metallization is the elemental analysis of thin barrier/seed layers through the depth of TSVs before and after Cu electroplating. The thin film characterization in the range of nanometers deep down into TSVs (within the range of hundred-micrometers) make it crucial to determine conformality, step coverage, purity and corrosion. With ToF-SIMS, we are able to check the corrosion under the influence of the Cu electrolyte [11] and map detailed elemental composition information onto the TSV wall.

In the second part, as an illustration, we choose to monitor the uniformity of silicon dopant concentration in the HfO_2_ thin films deposition as atomic layer. The recent discovery of ferroelectric properties in this binary oxide material [12], commonly established as a dielectric for high-k metal gate technology (HKMG), handover a vast quantity of applications beyond the standard dielectric material. These applications span from non-volatile memories [13,14], steep slope devices [15] energy storage applications [16,17] and infrared sensors [18]. In this part of study, we focus on improving the analysis of the HAR structures by bypassing the obstacles of TSV characterization by ToF-SIMS and using LHAR test structures. ToF-SIMS in dual-beam mode (dynamic mode) is a well-established technique due to its high detection sensitivity for concentration ratio measurements of the dopant materials [19,20]. However, the analysis of these VHAR structures for quantifying dopant concentration is hardly possible. To solve this issue, we analyzed layer composition in a different system while being consistent with the results of the initial structures. In this system, instead of using the vertically oriented structures, lateral high aspect ratio (LHAR) [21,22,23] structures are used which make it possible to analyze them in the form of 2D-structures.

## 2. Experimental

### 2.1. Materials and Thin Films Preparation

#### 2.1.1. TSV Metallization

The cobalt metal-organic chemical vapor deposition (Co-MOCVD) process enables manufacturing an adhesive and conformal thin film all the way through the TSVs, in comparison with the conventional non-conformal copper physical vapor deposition (Cu-PVD) process as a seed layer. However, the Co-MOCVD metallization mode requires achieving a proper seed layer with less organic contamination (e.g., carbon) which would intensify the Co corrosion under the Cu electrolyte influence. Due to the electroplating bath chemistry, and especially because of the Cu electrolyte component, the Co thin film is prone to corrosion during electrochemical deposition (ECD). The Cu ions content have great influence on Co corrosion. The redox reaction of Cu solidification is a fast corrosive factor. If the concentrations of the components are high enough in electrolyte, the cobalt corrosion reaction is faster than the Cu deposition. Due to this comparable faster dissolution of Co seed layer, there will most likely be non-copper-plated areas after the deposition process on the bottom of TSVs.

**Figure 1:**
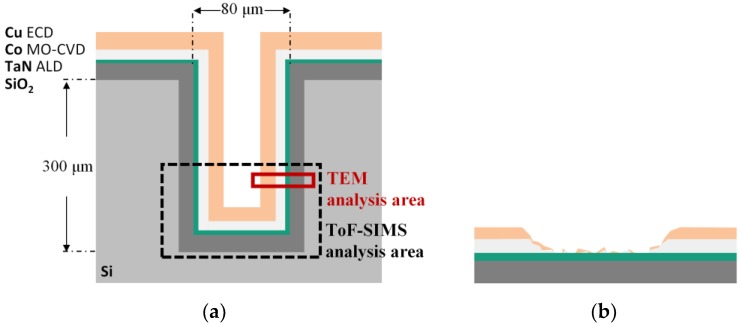
Schematic illustrations of (**a**) the ideal through-silicon-vias (TSV) metallization process after Cu electrochemical deposition (ECD) and (**b**) the corroded cobalt seed layer during Cu ECD.

Figure 1 schematically shows the structure (Figure 1a) and the corroded bottom sidewalls after electroplating (Figure 1b). With respect to the Co layer, the TSVs’ elemental and compositional depth profiles, especially on the bottom sidewall, were investigated before and after Cu-ECD. ToF-SIMS is used to measure the relative Co content in order to determine which combination of parameters yielded the highest and most stable amount of Co. The amount of Co ions result from ion gun sputtering through the depth of deposited films. Eventually, the comparative study with TEM was carried out to reveal the reliability of ToF-SIMS measurement.

Silicon wafers, sized 300 mm, with dry etched wholes with an aspect ratio of 1:4 were chosen to perform the metallization process. All barrier film stack depositions were carried out without breaking the vacuum of the cluster tool, from Applied Materials (AMAT) Endura 2. Tantalum-Nitride (TaN) was employed as the Cu diffusion barrier layer which was fabricated by the ALD process for achieving an excellent conformality all the way through the TSVs. Afterwards, the Co thin film was fabricated at a temperature of 150 °C using the Hexacarbonyl (3,3-dimethyl-1-butyne) dicobalt (CCTBA) precursor at 5 Torr by MOCVD. Subsequently, the ECD process was accomplished on the coupon level in a lab-scale plating cell right after a pre-wetting treatment. The plating cell was set up with a connected cathode (the structured sample) and an anode (Cu metal plate) electrodes which were immersed inside a low-copper electrolyte. The electrolyte contained copper ions and sulfuric acid at concentrations of 4 g/L and 10 g/L, respectively.

#### 2.1.2. HSO ALD Deposition

In this study, we used metal-organic Tetrakis (ethylmethylamido) hafnium (IV) (TEMAHf) and Tris (dimethylamino) silane (3DMAS) to form Si-doped HfO_2_ (HSO). Superior conformality of ALD thin film deposition is a direct consequence of the inherent self-limiting reactions [24]. The main goal for ALD process development for deep trench capacitors is to find precursors which allow a conformal deposition in HAR structures and show the same material composition along the trench sidewall. To achieve that, it is necessary to study the precursors’ behavior in deep trench structures. Due to the different partial pressures, molecular size and molar masses of different precursors, the gas diffusion behavior into the trenches will be different for processes using more than one precursor. Therefore, varying dopant concentration levels may occur throughout the depth of trenches.

**Figure 2:**
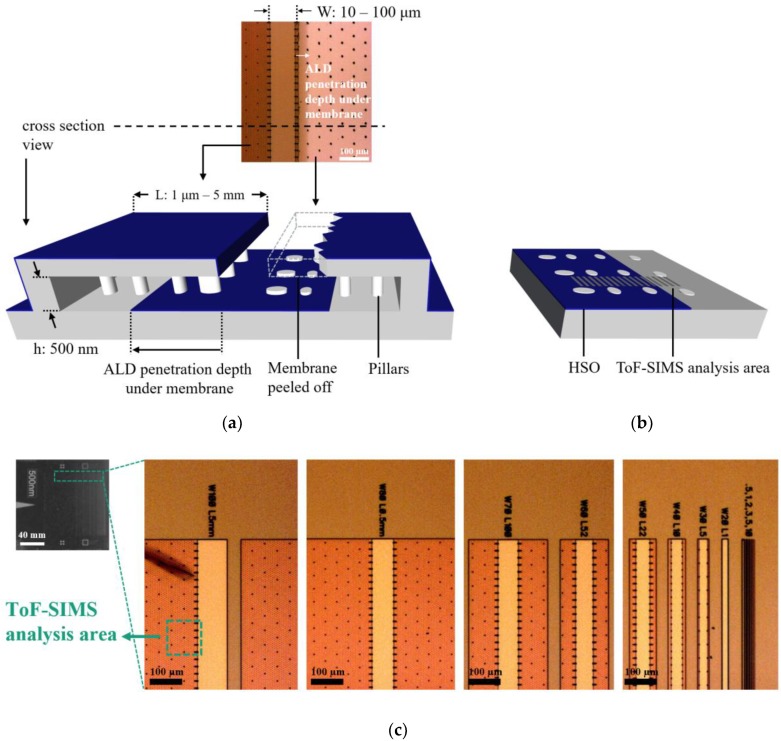
Schematic illustrations of (**a**) the PillarHall™ test structure (bottom) that is used in the atomic layer deposited (ALD) process, and the corresponding top-view optical microscopic image (top) and (**b**) the test structure after membrane removal. The diffusion depth of the ALD process can be examined directly by time-of-flight secondary ion mass spectrometry (ToF-SIMS). Color code: Gray = silicon substrate, Blue = deposited material with ALD, Dark gray rectangles = ToF-SIMS region of interests (ROIs). h: cavity height, W: opening width, L: lateral length of membrane (images dimension are not to scale). In (**c**), the uncoated test chip and the optical microscope images of the corresponding top-view from different cavities with the membrane is shown.

The absence of a simple and readily available 3D structure for elemental analysis of thin films produced by ALD led us to use a different system with the capability to analyze 3D HAR structures in the form of a 2D-planar surface. To be able to perform ToF-SIMS to optimize the deposition process, an LHAR structure is used, which is depicted schematically in Figure 2a. The microscopic LHAR structures were fabricated in chips on 150 mm silicon wafers using standard surface micromachining techniques. The chips contain multiple lateral cavities processed on top of single-crystal silicon with a polysilicon membrane roof sustained by polysilicon pillars [22]. The pillars provide a defined geometry with a nominal gap height of 500 nm. One chip contains LHAR structures (cavities) with different membrane lengths (L) from 1 μm to 5 mm (AR range 2:1–10,000:1), each with a single-crystal silicon area in front with defined width for easy identification (W = 100 µm, 90 µm, etc.). The roof of this LHAR test structure can be removed using adhesive tape and the deposited material can be assessed directly, as shown in Figure 2b. For thin film analysis with ToF-SIMS, we defined the analysis area at the 5 mm length membrane (W = 100 µm).

The HSO material was processed at a temperature of 280 °C using a thermal ALD process at a Jusung Eureka 3000 (Tokyo, Japan). The TEMAHf and O_3_ were used as a metalorganic precursor and oxidizer respectively for HfO_2_ deposition. For SiO_2_ doping, 3 DMAS/O_3_ cycles were inserted to achieve a controlled ratio of dopant and HfO_2_. During all processes, Ar gas was used for purging.

### 2.2. Analysis Tool Setups

The SIMS analysis data were acquired using a TOF-SIMS 300R (IONTOF GmbH, Münster, Germany) in order to analyze both overall film growth and doping levels inside the structures. The tool is equipped with a bismuth (Bi) liquid metal primary ion gun (LMIG). The primary ion source was used with a short pulse at the voltage of 25 kV (anode) at 45°. The beam was chopped in 20 ns and focused to about 300 nm in diameter. The ion beam current was ∼13 nA (DC mode). The secondary ions were collected with an extraction lens (biased at −40 V), traveled through the reflection flight tube and identified with a micro-channel plate (MCP) detector. A dose density of primary ions for each measurement was about 6.5 × 10^−13^ ions/cm^2^. The vacuum pressure in the main chamber was kept at about 1.0 × 10^−8^ mbar.

A mass spectrum for each pixel was collected by raster-scanning of the primary ion beam to obtain a mass spectrometry data image across the sample area of interest (150 × 150 μm^2^ using TSVs and 102 × 102 μm^2^ using LHAR test chips). An electron impact (EI) gas ion source of oxygen (O_2_^+^) was used as a second ion beam source to remove material for profiling electropositive elements in positive SIMS. The energy of the sputter gun was set to 1 kV to concede the image profile of interesting fragments in depth. The average beam current was 0.23 μA. The initial velocity of secondary ions was provided by an accelerator into the drift tube with a potential of 2 kV. This velocity distribution was implemented for the mass separation by flight time analysis. A mass resolution of 8300 (m/Δm) at 28 amu can be accomplished with the flow time transformed into atomic mass units (amu). For the image analysis, data were acquired from 400 × 400 μm^2^ by an ion beam raster of 256 × 256 pixels in a saw-tooth mode. Image analysis is conducted in SurfaceLab v6.3 (developed at IONTOF GmbH).

For step coverage analysis we used SEM by Hitachi S4800 (Hitachi, Japan) with an electron beam source energy at 10 kV using a back-scattered detector. For higher lateral resolution on the TSV cross-section, TEM observation was carried out for the structural analysis in localized areas at the bottom of the TSV sidewalls using FEI Tecnai F20 (Hillsboro, OR, USA). The acceleration energy of the electron beam was 200 keV. The process of lamella preparation for TEM observation is explained in Appendix A.

## 3. Results and Discussion

### 3.1. Vertical High Aspect Ratio Structures

**Figure 3:**
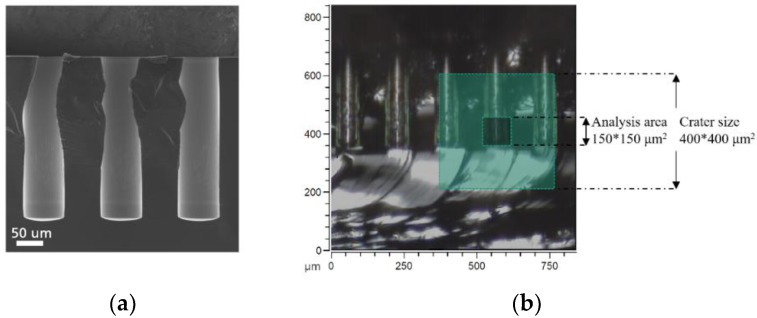
Cross-sectional images showing (**a**) an SEM micrograph of TSVs and (**b**) a camera snapshot of a mounted sample (without Cu plating) showing the side wall with the crater size and analysis area, as highlighted in the image, during the ToF-SIMS measurement.

As mentioned earlier, one of the problems with characterizing the elemental distribution in TSVs is the geometry of the structures in comparison with planar samples. The surface topography can cause undesirable artifacts in the SIMS image and spectra profile which significantly inhibits interpretation and quantification of data. As a consequence, surface evaluation and depth analysis with microscale topography are a considerable challenge due to analytical instrumental limitation. To study the inside of TSVs, the samples were cut and mounted on the sample holder in such a way that the cross sectioned TSV sidewalls face the analyzer. Figure 3 demonstrates the analysis area and sputter zone in the cross-sectional view.

**Figure 4:**
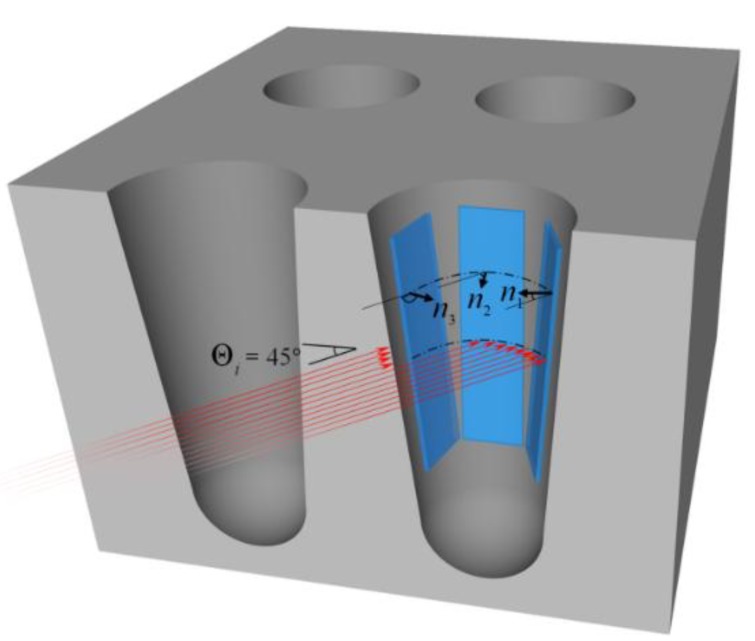
Schematic representation of a TSVs cross-section. Primary ion beams strike the sample’s top surface at an incident angle of 45° (red arrows). The *n*_1_, *n*_2_, and *n*_3_ denote the axes normal to each surface (blue colored planes). Each of these surfaces depict the relative angle between the incident ion beams and the inner surface of the TSV.

A 3D reconstruction of the ToF-SIMS data was used to map the elemental distribution. The resulting image intensity is influenced by the effect of sample surface orientation or surface topography of the analysis area on the trajectory of the primary ion beam. Sputter yields not only depend on the primary ion energy but also the angle of incidence [2,25,26]. The angle of incidence changes depending on of the surface topography [3]. This induces changes in the energy distribution of the emitted secondary ions, the sputtering yield, and the angular distribution of ejected fragments (based on the emission angle from the surface normal) [27,28]. This artifact can cause falsified variations in image intensities (see Figure 4).

**Figure 5:**
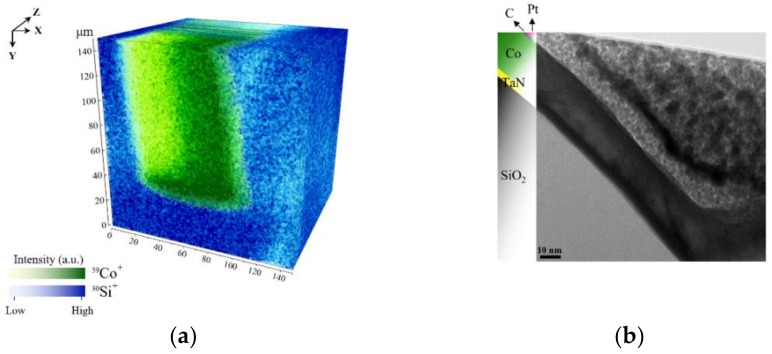
Images shown here are of the TSV before Cu electroplating. In (**a**) the 3D ToF-SIMS map of Si and Co signal from the bottom is shown and in (**b**) a TEM image from the bottom sidewall of the TSV showing the TaN barrier the cobalt film and the preparation fill.

**Figure 6:**
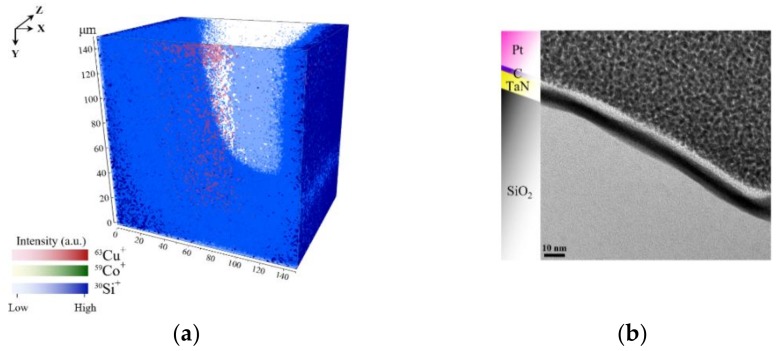
Images shown here are of the TSV after Cu electroplating. In (**a**) the 3D ToF-SIMS map of Si, Co and Cu signals from the bottom and in (**b**) a TEM image from the bottom sidewall of the TSV is shown.

The acceptance angle of the primary ions (red arrows) with respect to the sample normal of the left inner surface (*n*_3_), depicted in Figure 4, is limited in comparison to the middle and right inner surface (*n*_2_, *n*_1_). Consequently, the detection of secondary ions resulting from the right inner surface have a higher probability to reach the mass analyzer. Whereas, the secondary ions from the left inner surface can hardly reach the analyzer. The image profile of a 25 nm thick film of Co in the TSV sputtered using a 1 kV oxygen-ion beam, is shown in Figure 5a. Cobalt is visible as ^59^Co+ (*m*/*z* = 58.93) throughout the TSV. Figure 6a, shows that Co is completely corroded after electroplating. As illustrated in Figure 5a and Figure 6a, the same constructed layout, as in Figure 4, is built from ToF-SIMS analysis. The samples’ orientations, with respect to the primary ion beams, and the analyzer perfectly agree with the schematic representation in Figure 4.

In Figure 5a and Figure 6a, the geometrical influence on 3D imaging is pronounced for the trench sidewalls. A higher contrast is observed for Co and Cu of the sidewalls which face the primary ion gun. This confirms the influence of surface topography on SIMS 3D image reconstruction. In most SIMS instruments, the incident angle of the primary ions (viewing direction) creates an angle between 30° and 60°. As a result, the shadowing effects of the surface geometry are unavoidable [3]. Although, different approaches are suggested by Lee et al. such as sample alignment, changing extraction voltage, using cluster ion beam, and adjusting extraction delay to reduce the shadow effect [29]. However, this effect could not be eliminated entirely.

In addition, we compare the ToF-SIMS results with TEM micrographs which support the findings of ToF-SIMS shown in Figure 5b and Figure 6b. Both TEM lamellas are lifted out from the bottom of the TSVs. The Pt and C that are marked in the figures are used as the protection layers in the process of lamella preparation for TEM to protect the top surface from unwanted surface damage by FIB milling and amorphization with high energetic ions. Comparing the results from TEM with ToF-SIMS, 3D images determined the Co corrosion at the bottom of TSV. The TEM image in Figure 5b shows the 25 nm thick Co seed layer on top of the TaN, which matches with ToF-SIMS analysis results. After Cu electroplating, it is not possible to detect Co species at the bottom of the TSV sidewall. Besides, Cu signal intensity is very low, which supports the idea of complete corrosion at the bottom of the TSV. To check the precision of this observation, a new lamella was prepared from the sample after electroplating. The results in Figure 6b support the assumption of Co thin film corrosion and failure on Cu deposition.

### 3.2. Lateral High Aspect Ratio Structures

One type of ferroelectric based non-volatile memory is the 1T-1C ferroelectric random-access memory (FRAM) where the memory state is stored in a ferroelectric capacitor. The readout current through the access transistor becomes proportional to the ferroelectric remnant polarization. In order to improve the sense current, a common 3D-like structure is utilized where the area factor enhances the total polarization charge [30]. Various dopants influence the stabilization of the ferroelectric phase in hafnia thin films, for example, silicon (Si) [17,31,32], and such influence becomes more decisive in deep trench structures while the dopants concentration may change inside the HAR structure. Remarkably, it is more critical in low-doped materials, where such a low Si content is necessary, while a small deviation results in strong change in the stabilized phase [33]. As compared to planar film deposition, effects on the layer composition and crystal structure are expected to be different for HAR structures. Therefore, a technique to quantify the Si concentration in ferroelectric films deposited in 3D structures is crucial for optimizing the thin film deposition process of FRAM applications.

Due to the small dimensions of 3D structures it becomes crucial to reach an attainable lateral resolution of different analytical techniques. In ToF-SIMS 3D imaging, the dimension of collision cascade and surface damage define the limitation that one could reach in the lateral resolution. Consequently, the useful lateral resolution of SIMS imaging relies on the primary ion beam focus and ionization efficiency of the surface analysis [34]. In order to reach the maximal lateral resolution, it is a compromise between acquisition time (number of ions per shot), mass resolution (pulse length) and lateral resolution (spot size) is unavoidable. To succeed in high mass resolution, short pulses in the order of < 1 ns are required. Producing short pulses with higher energy cost deterioration of the focus in the range of 3–10 μm. In contrast, to achieve a lateral resolution in the range of 200–300 nm, it is required to increase the pulse length larger than 150 ns whilst decreasing the mass resolution. In spite of such a trade-off, it is also possible to simultaneously obtain high spatial and high mass resolution as described by Vanbellingen et al. by applying delay extraction of secondary ions [35].

**Figure 7:**
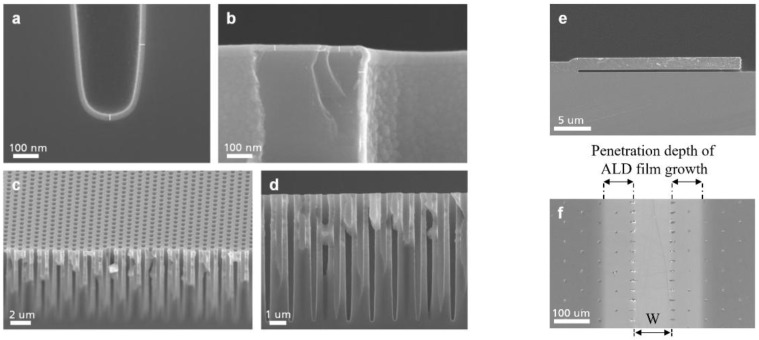
SEM cross-section of 20 nm Si-doped HfO_2_ (HSO) deposited on vertical high aspect ratio (VHAR) structures are shown in (**a**–**d**). In (**e**), a side-view SEM micrograph of PillarHall lateral high aspect ratio (LHAR) test structure (membrane width 20 μm) is shown and in (**f**), a top-view SEM micrograph of Si-doped HfO2 grown at 280 °C in LHAR structure (PillarHall) is shown. The membrane has been peeled off (membrane width > 500 μm). The central cavity opening for precursors flow shows with “W” (see Figure 2a). The diffusion depth of the ALD process can be examined directly by ToF-SIMS.

Figure 7a,b show SEM micrographs of VHAR structures with 20 nm of HSO deposited on them. The non-planar surface geometry of the collected samples’ cross sections influences the secondary ion yield and restricts the number of ions that can be created out of the sputtered area. In addition, the surface topography has an effect on the ultimate mass resolution, which arises from the slight differences in the extraction field [36]. In low-intensity imaging, many small features are affected by artifacts of counting statistics. This limits the total amount of achievable information and results in only a few number of counts of interest area per pixel which makes it crucial to properly evaluate the results from planer surfaces.

To improve image analysis, we facilitate the characterization procedure of HAR structures on the LHAR to create a suitable technique on imaging elemental distribution in non-planar structures with ToF-SIMS. Figure 7e,f illustrate an LHAR structure via SEM micrographs. The chips (PillarHall) can easily be brought on to a carrier wafer in the ALD chamber. After the deposition process, the membrane is peeled off using an adhesive tape.

**Figure 8:**
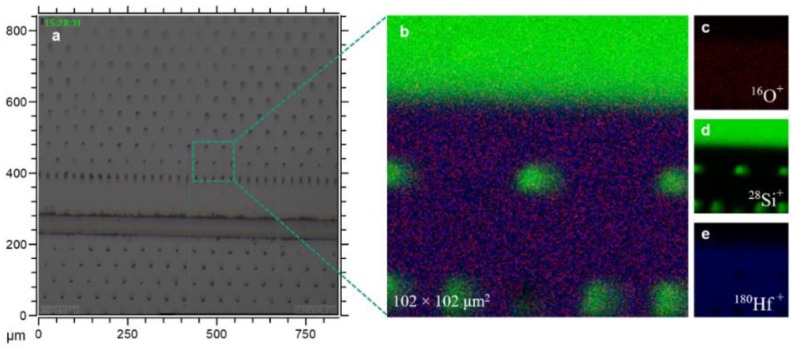
Images showing (**a**) snapshot using a microscope from the PillarHall after membrane removal (top view), (**b**) a superimposed image with ToF-SIMS of deposited region, and (**c**–**e**) an RGB representation of ^16^O^+^ (*m*/*z* = 15.994), ^28^Si^+^ (*m*/*z* = 27.978), and ^180^Hf ^+^ (*m*/*z* = 179.949).

**Figure 9:**
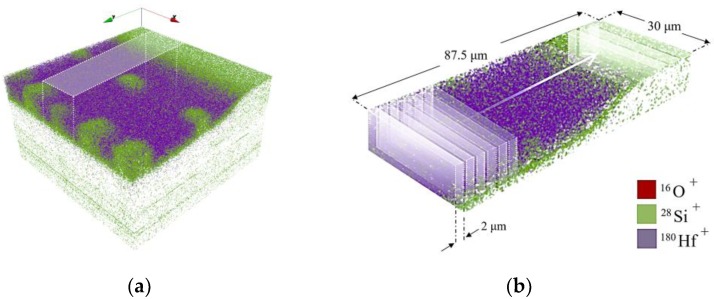
(**a**) A volumetric view of the distribution of ^16^O^+^ (*m*/*z* = 15.994), ^28^Si^+^ (*m*/*z* = 27.978) and ^180^Hf^+^ (*m*/*z* = 179.949) over a 102 × 102 μm^2^ field of view from the deposited region after peeling off the membrane. (**b**) A defined ROI with dimension of 2 × 30 µm^2^ to integrate total counts of the interested area from Si and Hf.

**Figure 10:**
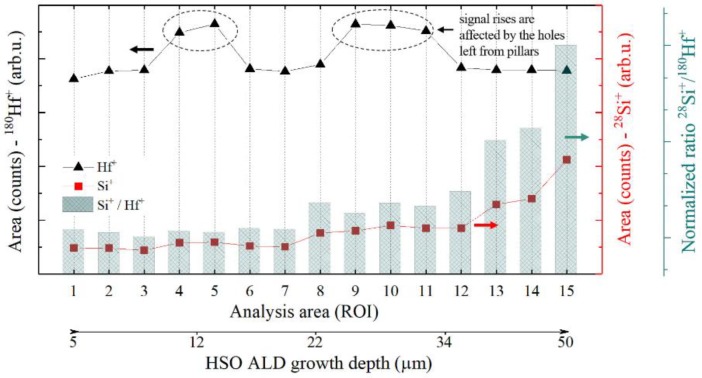
The ^28^Si^+^, ^180^Hf^+^ and the ratio signals of the HfO_2_ layer obtained by ToF-SIMS analysis of the LHAR test structure with respect to the diffusion depth.

Figure 8b represents the total ion image spectrum of the acquired area associated with the ^16^O^+^, ^28^Si^+^, and ^180^Hf^+^ signals. In Figure 8c–e, each elemental signal is color coded in red, green and blue for ^16^O^+^, ^28^Si^+^, and ^180^Hf^+^, respectively. The green dots in Figure 8b,d are the holes left by the pillars after membrane removal. Figure 9a illustrates the volumetric view by 3D data analysis of the distribution of ^16^O^+^ (*m*/*z* = 15.994), ^28^Si^+^ (*m*/*z* = 27.978) and ^180^Hf^+^ (*m*/*z* = 179.949) over a 102 × 102 μm^2^ area. To assess the deposited material in the LHAR structure with respect to the depth, the region of interests (ROIs) with a dimension of 2 × 30 µm^2^ were defined as depicted in Figure 9b. For each ROI, the ^28^Si^+^ and ^180^Hf^+^ species total counts from emitted secondary ions are integrated, where the influence of the silicon substrate is excluded based on the ^28^Si^+^ depth profile variation from 16% to 84% on minimum ^28^Si^+^/^180^Hf^+^ ratio (Figure 10_ROI.01). We are assuming a homogeneous spatial distribution of Si^+^ and Hf^+^ on each ROI (2 × 30 μm^2^).

The ToF-SIMS analysis results of the HSO material in the LHAR structure are shown in Figure 10. The integrated counts for the ^180^Hf^+^ and ^28^Si^+^ species are plotted with respect to the depth inside the test structure. Due to the geometry of the sample, the analysis starts at a minimal distance of 5 µm from the inlet slit. The absolute intensity is not constant as the sputter rate exhibits local variations (not shown here). However, the ratio of the ^180^Hf^+^ and ^28^Si^+^ signals is consistent over a depth range from 5 µm to 30 µm.

Total area counts of the ^28^Si^+^ and ^180^Hf^+^ signals were calculated from the defined areas (ROIs) of the 3D reconstruction (as seen in Figure 9b). A constant ^28^Si^+^ to ^180^Hf^+^ signal ratio is observed over the depth range until *c.a.* 30 μm (deviation of 5%) which corresponds to an aspect ratio up to 1:50 with uniform dopant concentration. At a depth higher than 35 µm, the Si content rises. This can be due to the Si substrate influence. However, up to *c.a.* 30 μm, the step coverage (or depth penetration) of 3DMAS/O_3_ (SiO_2_) is as good as TEMAHf/O_3_ (HfO_2_) at the chosen process conditions.

## 4. Conclusions

This study aimed to illustrate a novel method to address the challenges of elemental analysis in high aspect ratio structures and to provide a practical guideline on the effect of surface topography on ToF-SIMS. As shown, the main difficulty in the interpretation of non-planar structures is to reach optimal material removal and sensitivity for a given material. As shown in the first study, on the vertical TSVs, the image intensity is affected by the surface topography and results in an inaccurate representation of 3D imaging. Since the sputter yield is a function of the primary ion beam incident angle (incident at θ = 45° to the sample normal for a flat sample), the geometrical shadowing effects are inevitable. In addition, we used lateral high aspect ratio (LHAR) test structures (PillarHall™) as a new platform to perform precise measurements of hafnia doped thin films. In contrast to the common vertical HAR structures, the combination of ToF-SIMS 3D imaging and LHAR test chips leads to a constructed footprint of the composition of the deposited thin film without being influenced by the sputter yield and the surface topography. By integrating the total data points of the ROIs, semi-quantitative calculations on the ratio of elemental distribution over the depth of the LHAR structures were calculated. In conclusion, surface topographic effects could be eliminated by using LHAR structures, allowing the separation of chemical variations within the analysis area. Further investigations are planned to precisely compare the stoichiometry of the deposited material in the planar structures with the deposited film in the depth of the LHAR structures by coupling results from ToF-SIMS and X-ray photoelectron spectroscopy (XPS).

## Figures and Tables

**Figure A1:**
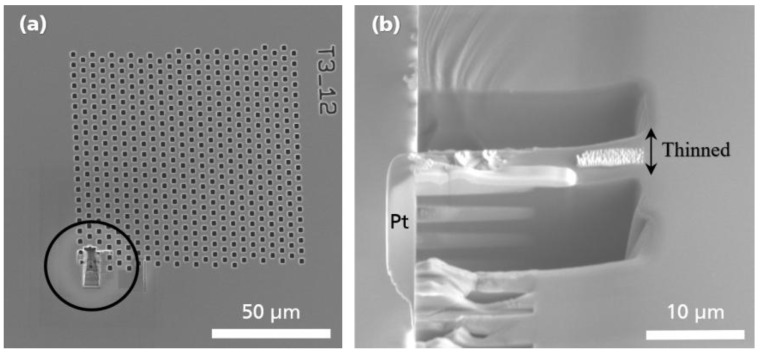
SEM images of (**a**) structure layout and (**b**) SEM images of applied carbon and e-Pt protection layers to the cleaved TSV and rough thinning lamella before lift-out and after tilting.

**Figure A2:**
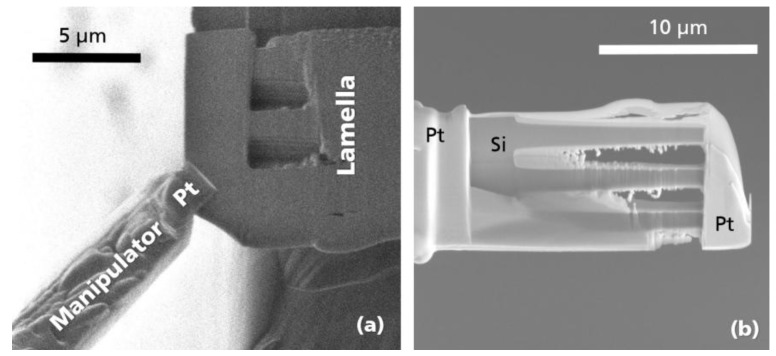
SEM images of (**a**) lifting out the free lamella and (**b**) oblique view during finishing and post-finishing processes with i-Beam and e-Beam.

**Figure A3:**
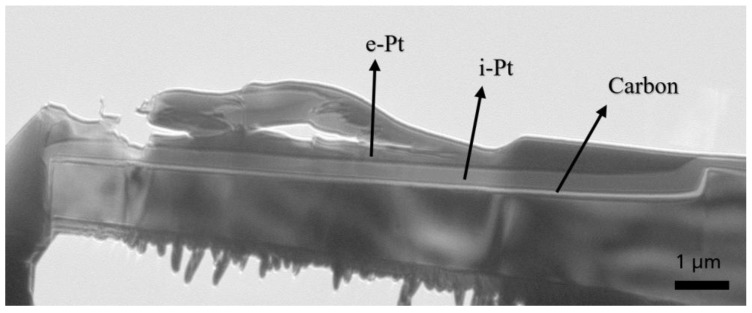
TEM images of the entire overview of halved TSV structure.

## Appendix A

The main challenge of TSV characterization is to gain information from inside of the hole particularly the bottom part. As the TSV dimensions are almost larger than the cutting methods capability, such as FIB cross sectioning, it is hard to design a metrology in order to investigate the depth compositional profile, layer thicknesses, surface quality, conformality and overall step coverage all the way through a hole. The FIB could perform on TSV, but there are some challenges involved, such as sidewall Si redeposition, time consumption in the range of hours as well as low accuracy of a wavy cut known as curtaining effect. Transmission electron microscopy (TEM) with high resolution images gives the most information about deposited layers in a few nanometers range, but it takes lot of time and effort, not only for sample imaging, but also for sample preparation.

According to the aforementioned challenges, for observing the TSV bottom sidewalls with TEM, the sample was cleaved precisely. Therefore, a series of indentations before and after the target structure were applied by a Nano Indenter tool in a way to hit the TSV. Subsequently, the cleaved sample was transferred to the FIB/SEM Strata 400, FEI tool. Figures below show the lamella preparation and lift-out procedure systematically using FIB technique. Carbon and e-Pt protection layers were applied respectively before utilizing any ion beam so as to protect the thin film on the structure (Figure A1a).

After depositing carbon, e-Pt and also i-Pt protection layers, the target layer is safe, and it is time to dig in the lamella surroundings. Then, as the lamella is still attached and stable enough, rough thinning has to be done line by line via “cleaning cross section thinning” mode before lift-out (Figure A1b). After the lamella release-cutting at the very bottom, next step is lifting out the free lamella which was welded earlier with Pt to the Omniprobe manipulator (Figure A2a) by GIS.

Then, the lamella was welded to the TEM sample holder. The manipulator welded area was unattached and finally it was withdrawn. In this stage, finishing and post-finishing processes of the lamella with i-Beam and e-Beam were performed respectively which is shown in Figure A2b. The sample is now thin and transparent enough for TEM observation. Therefore, the welded sample to the sample holder was transferred to the TEM Tecnai F20 200 kV, FEI tool for high resolution pictures (Figure A3).
